# Chlorinated Water Induced Aging of Pipe Grade Polypropylene Random Copolymers

**DOI:** 10.3390/polym11060996

**Published:** 2019-06-04

**Authors:** Joerg Fischer, Paul J. Freudenthaler, Reinhold W. Lang, Wolfgang Buchberger, Susan C. Mantell

**Affiliations:** 1 Johannes Kepler University Linz, Altenberger Strasse 69, 4040 Linz, Austria; paul.freudenthaler@jku.at (P.J.F.); reinhold.lang@jku.at (R.W.L.); wolfgang.buchberger@jku.at (W.B.); 2 Department of Mechanical Engineering, University of Minnesota, 111 Church Street SE, Minneapolis, MN 55455, USA; smantell@umn.edu

**Keywords:** polypropylene, pipe grades, chlorinated water, aging, fatigue crack growth resistance, superimposed mechanical-environmental testing

## Abstract

Polypropylene random copolymers (PP-R) are common materials for pressurized hot water pipes. In many pipe systems, potable water is disinfected by chlorine to prevent waterborne diseases. This paper deals with hot chlorinated water induced aging of two PP-R grades with varying morphology. One material had a conventional monoclinic α crystal form (PP-Rα), whereas the other was explicitly beta-nucleated resulting in a trigonal β crystal form with a fine spherulite structure (PP-Rβ). Micro-sized specimens with a thickness of 100 µm were used for aging experiments at 60 °C in chlorinated water with 5 mg/L free chlorine, and aging indicators were monitored for exposure times of up to 2000 h. On the other hand, superimposed mechanical-environmental tests were carried out by using cracked round bar specimens with a diameter of 14 mm to determine the fatigue crack growth (FCG) resistance of both PP-R grades at 60 °C in non-chlorinated and chlorinated water. PP-Rβ was found to outperform PP-Rα with an about 30% higher time-to-embrittlement value of 2000 h. Furthermore, PP-Rβ exhibited an enhanced FCG resistance in both non-chlorinated and chlorinated water. The effect of chlorine content on the deterioration of the FCG resistances was significantly more pronounced for PP-Rα.

## 1. Introduction

Plastic pipes and fittings in pressurized pipe systems for hot water applications with temperatures up to 60 °C represent an important market for plastics pipes and are usually produced from polypropylene random copolymers (PP-R) [1,2,3]. Material properties in PP-R for such applications are typically tailor-made, and the morphology is adjusted via copolymerization with α-olefins or by incorporating specific additives. An increase of co-monomer content and/or the addition of nucleating agents results in smaller crystallite sizes and a finer spherulitic structure [1,4]. This leads to an enhanced density of trans-spherulitic tie molecules and inter-spherulitic entanglements and, in turn, to improved impact toughness and crack growth resistance. According to the standard ISO 15874, the high-performance pipe material class PP-RCT is defined as a polypropylene random copolymer with distinct crystalline morphology and improved pressure and temperature resistance, allowing for the production of pipes for higher pressures or with thinner wall thicknesses at constant loads [2,3]. Indeed, beta-nucleated PP-R with its trigonal β crystal form and its fine spherulite structure is classified as PP-RCT material, and it is recommended for pressure pipes in hot water circulation systems and for other applications where materials with excellent impact strength, chemical resistance and crack growth resistance are required [1,4,5,6,7,8,9].

Tap water is often disinfected to prevent waterborne diseases [10]. Therefore, various water treatment methods are in operation. These range from membrane treatments to ultraviolet irradiation and include the utilization of different oxidative chemicals. However, the latter category and more specifically the treatment with chlorine represents the most common disinfection method [10]. For drinking water, chlorine contents of up to 5 mg/L free chlorine and a pH between 6.5 and 7.6 is typically recommended [10,11]. Moreover, and in addition to disinfection, temperatures of about 60 °C are suggested for hot water applications to prevent growing and spreading of legionella [12].

The oxidative nature of disinfectants is known to affect the performance of polymeric materials in pipe applications, especially when operated at elevated temperatures and pressures [13,14,15,16,17,18,19,20,21,22,23]. In fact, numerous reports exist on accelerated aging and on premature failures of hot water polyolefin pipes exposed to chlorinated water (i.e., chlorine and chlorine dioxide) [17,24,25,26,27,28,29]. Thus, for polyolefins, strong evidence exists that the presence of disinfectants results in accelerated material aging. Simultaneously, so far very little knowledge is available on the precise nature of these accelerated aging mechanisms. Furthermore, the simultaneous exposure (i.e., superposition) to mechanical stresses and aggressive environments has a deteriorating effect on material performance and hence also acts to reduce pipe lifetimes [13,14,15,16,19,20,30,31]. Combined mechanical-environmental loading has been proposed to induce local molecular degradation at inherent defects of pipes, leading to accelerated crack growth and premature pipe failure [13,14,15,16,32,33].

Considering the practical importance of chlorine-induced premature failure of PP pipes and the lack of understanding of the underlying molecular and morphological mechanisms, it is the objective of this paper to investigate such global and local aging phenomena in two PP-R pipe grades of differing semi-crystalline morphology when exposed to hot chlorinated water at 60 °C, and to describe and discuss the effects of aging on the mechanical performance of these materials. Two test series were carried out, one aiming at the determination of aging indicators using micro-sized specimens that were pre-exposed in hot chlorinated water for various times and subsequently tested for their tensile performance, the other one investigating the crack growth resistance of these materials under superimposed mechanical-environmental cyclic loading.

## 2. Experimental

### 2.1. Materials

For all experiments, two commercially available polypropylene random copolymers (PP-R) were used. Both materials are PP-R pipe grades of high mean molar mass but differing semi-crystalline morphologies. The crystalline phase of one material is of the monoclinic α crystal type (PP-Rα) and the other a trigonal β crystal type (PP-Rβ) with small crystallites and a particular spherulite structure [1,4]. The material designations together with information on the crystal phase and pipe material classification are summarized in Table 1.

### 2.2. Specimens

**Figure 1:**
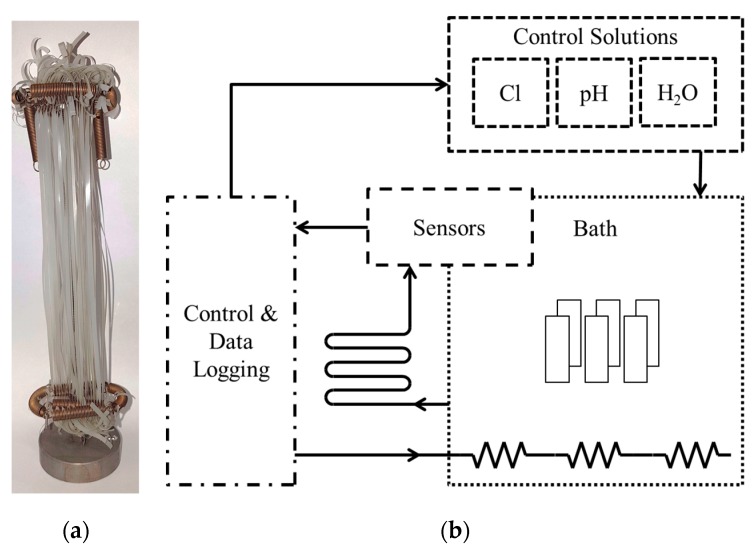
(**a**) Micro-sized specimens mounted on a unique device for immersion in chlorinated water; (**b**) illustration of the control scheme of the chlorinated water bath.

**Figure 2:**
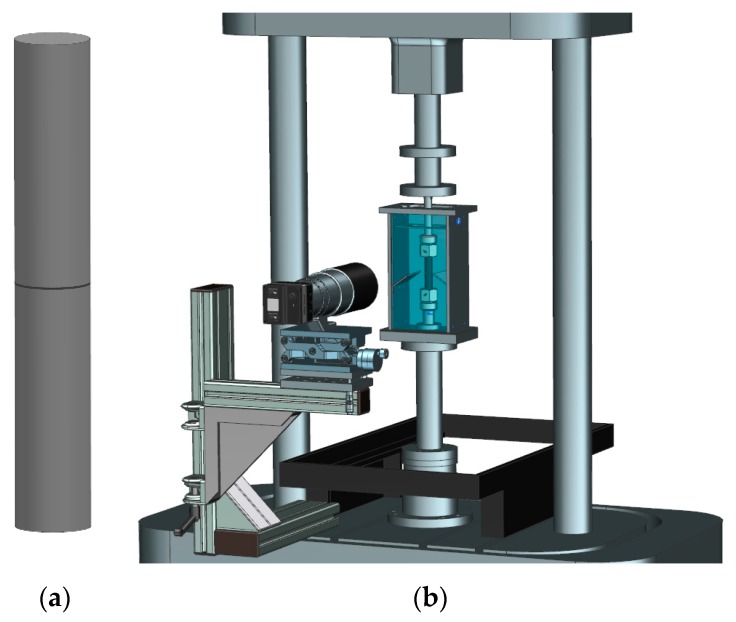
(**a**) Schematic illustration of a cracked round bar (CRB) specimen; (**b**) CRB specimen mounted on a tension-torsion electro-dynamic testing machine equipped with an environmental containment and an optical crack length measurement device.

To investigate the effect of hot chlorinated water on the material degradation behavior and the resulting mechanical tensile properties, micro-sized specimens [23,34] (Figure 1a) were produced out of injection molded plaques (Engel Victory 60, Schwertberg, Austria) via planing with the four-axis milling machine EMCO Mill E600 (EMCO, Hallein, Austria). The nominal specimen dimensions were 150.0 × 2.0 × 0.1 mm (length × width × thickness). Moreover, the superimposed mechanical-environmental cyclic tests were conducted with cracked round bar (CRB) specimens [35] with a diameter of 14 mm (Figure 2a). The CRB specimens were manufactured from 15 mm thick compression molded plaques with the lathe of the type EMCO 14D (EMCO, Hallein, Austria). The initial circumferential crack with a crack length of 1.5 mm was cut with a razor blade mounted on the machine via a razor blade holder.

### 2.3. Pre-Conditioning of Micro-Sized PP-R Specimens for Monitoring of the Aging Behavior

To monitor any aging effects of PP-R in hot chlorinated water, micro-sized specimens were pre-exposed for various times prior to analytical and mechanical characterization. A unique non-corrosive device was used to mount and immerse specimens in a water bath with controlled parameters (Figure 1) [22,23,36]. Chlorine content, temperature, and pH were set to 5 mg/L free chlorine, 60 °C, and 7, respectively. Specimens were removed after 250 h intervals for up to a total exposure time of 2000 h for further characterization.

### 2.4. Test Methods for Monitoring the Aging Behavior of Micro-Sized PP-R Specimens

The test methods and techniques for characterizing any material aging by various aging indicators included analytical techniques such as high-pressure liquid chromatography coupled with ultraviolet spectrometry (HPLC-UV), differential thermal analysis (DTA), gel permeation chromatography (GPC), infrared (IR) spectroscopy, and thermogravimetric analysis (TGA). Ultimate mechanical properties were determined via tensile tests of micro-sized specimens.

HPLC-UV was used to investigate the consumption of the non-oxidized phenolic antioxidants Irganox 1330 and Irganox 1010 (BASF, Ludwigshafen, Germany) as a function of exposure time [37]. Measurements were carried out with an HPLC of the type 1260 Infinity (Agilent, Santa Clara, CA, USA) equipped with a Kinetex C18 separation column (Phenomenex, Torrance, CA, USA) and a UV-detector. Tributylophosphite was added to avoid stabilizer loss during sample preparation, and Irganox L109 (BASF, Ludwigshafen, Germany) was utilized as an internal standard.

Differential thermal analysis was performed to determine the exposure time-dependent oxidation induction temperature (dynamic OIT) [38]. Perforated aluminum pans were used to test samples with the differential thermal analyzer DSC 4000 (PerkinElmer, Waltham, MA, USA) in the temperature range from 23 to 300 °C, and with a heating rate of 10 K/min in synthetic air. Samples with a weight of about 5 mg were cut from non-exposed and exposed micro-sized specimens.

GPC measurements were carried out to obtain mean molar masses (*M*_w_) and molar mass distributions of the non-exposed and exposed micro-sized specimens. Therefore, a high-temperature gel permeation chromatograph (PolymerChar, Valencia, Spain) equipped with an IR 5 detector was used. Samples were dissolved in trichlorobenzene, and heptane was added as a flow marker. The GPC was calibrated with polypropylene standards.

IR spectroscopic analysis was done by attenuated total reflection (ATR) mode to establish the carbonyl index (CI). Specimens were analyzed with an infrared spectrometer of the type Spectrum 100 (PerkinElmer, Waltham, MA, USA). The CI values were evaluated from the ratio of CO-stretching peak 1715 cm^−1^ and CC-stretching peak 974 cm^−1^.

Tensile tests were performed to determine the exposure-dependent strain at break values [39]. Therefore, the universal testing machine Instron 4202 (Instron, Norwood, MA, USA) was utilized. It was equipped with a 100 N load cell, and the tensile tests were conducted at 23 °C with a gauge length of 20 mm and a test speed of 50 mm/min. Average strain at break values were evaluated from the results of tensile tests with five specimens per exposure time.

### 2.5. Superimposed Mechanical-Environmental Fatigue Tests

The effect of superimposed mechanical-environmental loading in chlorinated water on the performance of PP-R pipe grades was investigated via fatigue crack growth (FCG) experiments. CRB specimens were tested with the tension-torsion electro-dynamic testing machine ElectroPuls E10000 (Instron, Norwood, MA, USA) equipped with a self-developed environmental containment (Figure 2) [14,16]. The containment was connected to a temperature and chlorine control unit to allow for tests at 60 °C in non-chlorinated (0 mg/L free chlorine) and chlorinated (5 mg/L free chlorine) water with a given pH of 7 [14]. For quasi-automatic optical crack length measurements, the testing machine was equipped with a crack length measurement device containing of an LXG-120M camera (Baumer, Frauenfeld, Switzerland), a Micro-Nikkor AF 200 mm f/4 D ED lens (Nikon, Tokyo, Japan), an LED flash RT STROBE 3000 (Rheintacho, Freiburg, Germany), and a self-programmed computer software for image capturing and processing.

For the FCG tests, specimens were loaded with a sinusoidal force with a frequency of 10 Hz and an R-ratio (ratio between the minimum and maximum applied stress) of 0.1. The applied maximum forces were selected allowing for quasi-brittle crack growth over a broad regime of crack growth rates and for practical testing times.

Values for the stress intensity factor range Δ*K*, describing the local cyclic stress field at the crack tip, were calculated by using Equations (1) and (2) for CRB specimens [40], where *“∆F”* represents the applied sinusoidal force range, *“r”* the radius of the CRB specimen, *“a”* the crack length, and *“b”* the radius of the initial ligament (*b = r − a*).

(1) $$\Delta K=\;\frac{\Delta F}{\pi .b^{2}}\sqrt{\frac{\pi .a.b}{r}}.f\left(\frac{b}{r}\right),$$

(2) $$f\left(\frac{b}{r}\right)=\frac{1}{2}.\left(1+\frac{1}{2}.\left(\frac{b}{r}\right)+\frac{3}{8}.{\left(\frac{b}{r}\right)}^{2}-0.363.{\left(\frac{b}{r}\right)}^{3}+0.731.{\left(\frac{b}{r}\right)}^{4}\right).$$

Crack growth rate values, d*a*/d*N*, were calculated from average crack length data by using a secant procedure [41]. FCG curves are presented in double-logarithmic plots depicting the d*a*/d*N* as a function of the applied stress intensity factor range Δ*K*. In terms of performance, enhanced FCG resistance is reflected by an FCG curve shift to lower crack growth rates and to higher Δ*K* values, respectively [42,43,44].

To illustrate the fracture surface appearance, microscope images of the CRB fracture surfaces were taken with the laser scanning confocal microscope (LSCM) LEXT OLS 4000 (Olympus, Tokyo, Japan). For the LSCM intensity images, the fracture surfaces were analyzed by layered scanning with a 405 nm laser. A microscope objective with a magnification of 20× was used, and the individual surface images were stitched by software to observe the whole fracture surface. In the LSCM images, the brightness of the image correlates with the roughness of the surface. Thus, darker regions in the image represent rougher surfaces. Moreover, a scanning electron microscope (SEM) of the type JEOL 6400 (JEOL, Tokyo, Japan) was applied to obtain higher magnification images of the quasi-brittle crack growth region of the fracture surfaces. For the SEM images, the fracture surfaces of the CRB specimens were sputtered with a thin gold layer to increase the surface conductivity.

## 3. Results

### 3.1. Effect of Chlorinated Water on the Aging Behavior of Micro-Sized PP-R Specimens

**Figure 3:**
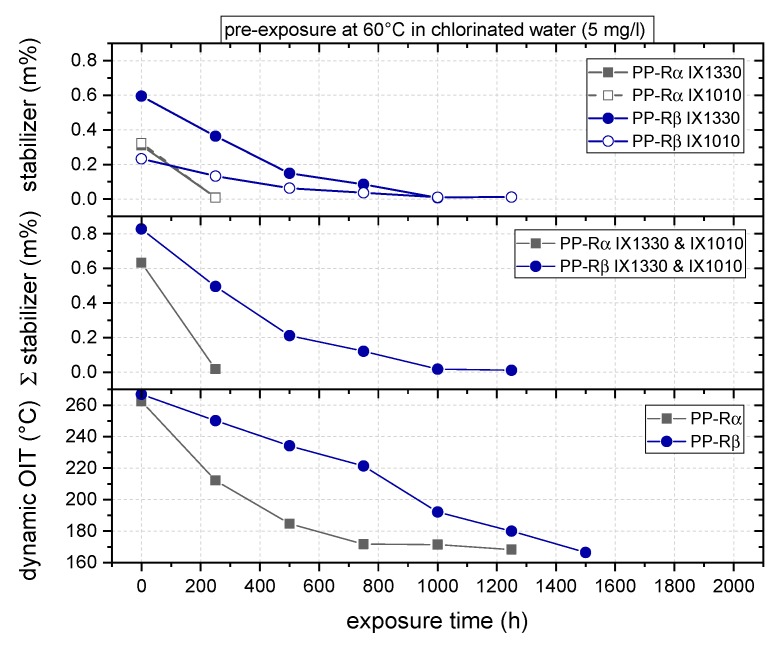
Phenolic antioxidants (IX1010 and IX1330) content and oxidation induction temperature (dynamic OIT) as a function of exposure time for the two polypropylene random copolymers (PP-R) grades.

Comparing PP-Rα and PP-Rβ, Figure 3 illustrates the aging-induced decline of the primary phenolic antioxidants IX1330 and IX1010 over the exposure time. Furthermore, the decrease in oxidation induction temperature (dynamic OIT) is shown, also as a function of exposure time. Starting with the evolution of the IX1330 content, a substantial reduction with increasing exposure time is apparent for both materials. However, the time for total stabilizer consumption/extraction with 250 h for PP-Rα is significantly lower compared to 1000 h for PP-Rβ. Similar deterioration characteristics were found for the phenolic antioxidant IX1010. While for PP-Rα again a total stabilizer loss was observed after 250 h, the corresponding value for PP-Rβ is 1250 h.

As to the total stabilizer content, it is evident that PP-Rβ with 0.83 m% comprised a higher initial content compared to PP-Rα with 0.63 m%. The significantly longer time to total stabilizer loss in PP-Rβ is most likely due to the finer spherulite structure, thus hindering the extraction capability in the polymer and decelerating the local interaction of the oxidization-inducing chlorinated water with the amorphous regions in the PP-R grade and therefore the stabilizer consumption. In this context, it is worthwhile mentioning that polymer additives in semi-crystalline polymers were found to be preferentially situated at the interfaces of spherulites [45].

Looking at the dynamic OIT data, with 263 and 267 °C for PP-Rα and PP-Rβ, respectively, both materials exhibited comparable initial dynamic OIT values. However, the higher number of total antioxidants in the beta-nucleated PP-R is again reflected by a slightly higher dynamic OIT value. In PP-Rα, the decrease in oxidation induction temperature is more pronounced in the first period of exposure, expressing in a drop of approximately 80 °C to a dynamic OIT of about 185 °C after 500 h followed by a slight drop to about 165 °C after an aging time of 1250 h. At higher exposure times, the dynamic OIT of PP-Rα was found to approach the melting peak, which prevented any further oxidation induction temperature evaluation. Conversely, the exposure of PP-Rβ to chlorinated water resulted in a continuous decline of dynamic OIT values, with the melting temperature being approached after about 1500 h. For both materials, the dynamic OIT decline is in good agreement with and corroborates the decrease in stabilizer content.

**Figure 4:**
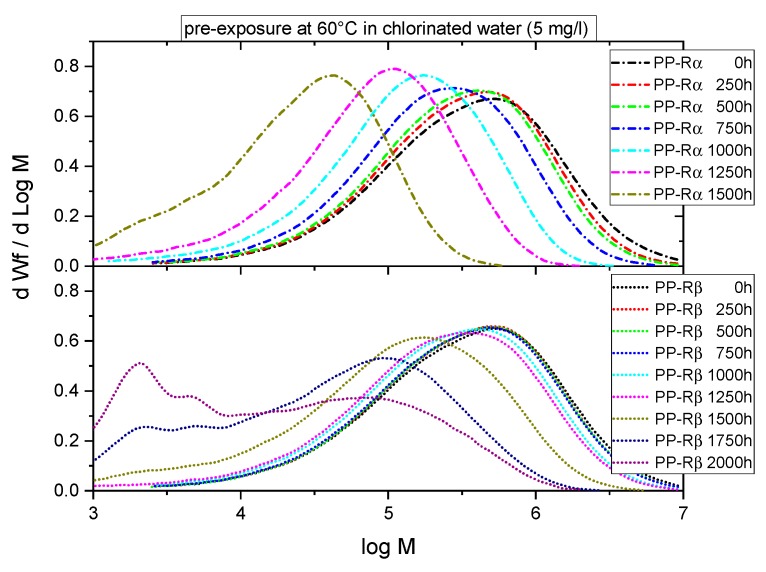
Time-dependent molar mass distribution for the two PP-R grades.

Figure 4 illustrates molar mass distribution curves of the two PP-R grades for exposure times ranging from 0 h (unexposed reference state) to 1500 h for PP-Rα and to 2000 h for PP-Rβ. Clearly, and especially for the alpha-nucleated PP-R, the number of long chain molecules is continuously reduced as the exposure time increases. These changes in the range of the higher molar mass values are seen even after 250 h of exposure, with significant shifts and overall shape changes of the molar mass distribution curves becoming apparent after 750 and 1500 h, respectively. The latter changes indicate a pronounced decline in mean molar mass, thus exhibiting a reduction in the extent of entanglements and in the number of tie molecules, which connect the crystalline structures ranging from crystalline lamellas to spherulites.

When comparing PP-Rα and PP-Rβ, the initial changes in the molar mass distribution up to 1500 h are significantly less pronounced for the latter material. Moreover, for longer times, when PP-Rα is already degraded to total embrittlement, the molar mass distribution for PP-Rβ transforms into a multimodal distribution with an increasing fraction of low molar mass content below 10 kg/mol and reaching a state of total embrittlement after 2000 h.

**Figure 5:**
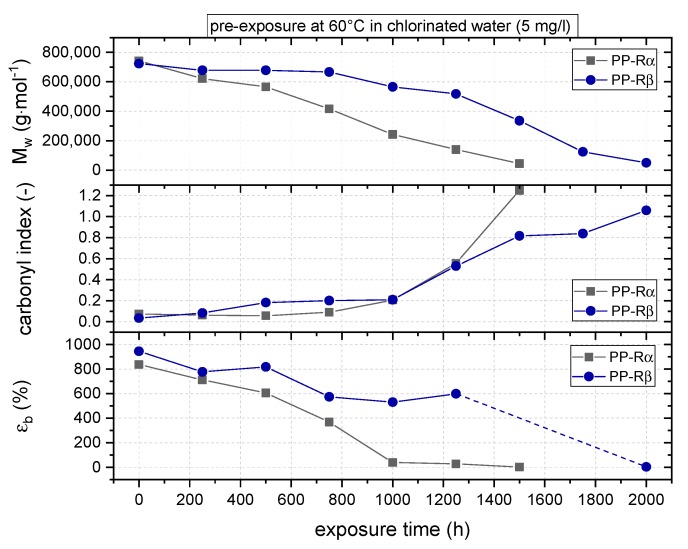
Molecular mass (*M*_w_), carbonyl index, and strain at break (ε_b_) as a function of exposure time for the two PP-R grades.

To elucidate a correlation between analytical aging indicators and aging-induced mechanical performance degradation, the evolution of the data for mean molar mass (*M*_w_), carbonyl index (CI), and strain at break (ε_b_) as a function of exposure time is depicted Figure 5. The initial mean molar mass values for PP-Rα and PP-Rβ are 742 and 723 kg/mol, respectively. *M*_w_ of PP-Rα is seen to drop continuously with increasing exposure time, reaching a mean molar mass of about 45 kg/mol after 1500 h. Hence, significant chemical aging processes and material degradation occur already in the earliest stages of exposure and continuously evolve further until total embrittlement is reached after 1500 h. Conversely, for PP-Rβ, the mean molar mass in the initial exposure time drops only slightly to 670 kg/mol after 750 h. Between 750 and 1750 h exposure, the decline in *M*_w_ is more pronounced, followed by a final drop to a mean molar mass of 50 kg/mol after 2000 h.

By comparison, the initial CI values of about 0.1 for both PP-R grades increased only slightly to 0.2 after 1000 h of hot chlorinated water exposure. Subsequently, significantly higher carbonyl indices were obtained at the surfaces of both materials, with maximum values of higher than 1.0 after 1500 and 2000 h for PP-Rα and PP-Rβ, respectively. Apparently, CI determination is much less sensitive to detect molecular degradation effects than GPC and perhaps dynamic OIT measurements.

Finally, corroborating the mean molar mass evolution, a continuous decrease in strain at break was obtained for both materials over the exposure time. Again, the reduction in strain at break values over exposure time is much more pronounced for PP-Rα, resulting in a reduction from initially 840% to about 40% after 1000 h of exposure, with a further reduction to 3% after 1500 h. This latter strain at break value is below the yield strain value of 18% of the unexposed material state of PP-Rα, thus indicating large-scale material embrittlement. By comparison, the initial strain at break of 950% for the non-exposed PP-Rβ decreased to a relatively high strain at break of about 530% after 1250 h of exposure. For the exposure times 1500 and 1750 h, only one specimen was tested to keep the remaining specimens for longer exposure times, as there was no indication of reaching total embrittlement yet. Strain at break values in these one specimen experiments were found to be still significantly above the yield strain value of 18% of the non-exposed micro-sized PP-Rβ specimens. After 2000 h of exposure, five micro-sized specimens were tested as the mean value for strain at break dropped to 4%, which represents total embrittlement of PP-Rβ. With this tendency for more post-yield deformation capability even after long exposure times, based on the tensile behavior, the beta-nucleated PP-R implies a higher resistance to hot chlorinated water compared to PP-Rα.

### 3.2. Effect of Chlorinated Water on the Fatigue Crack Growth Resistance

**Figure 6:**
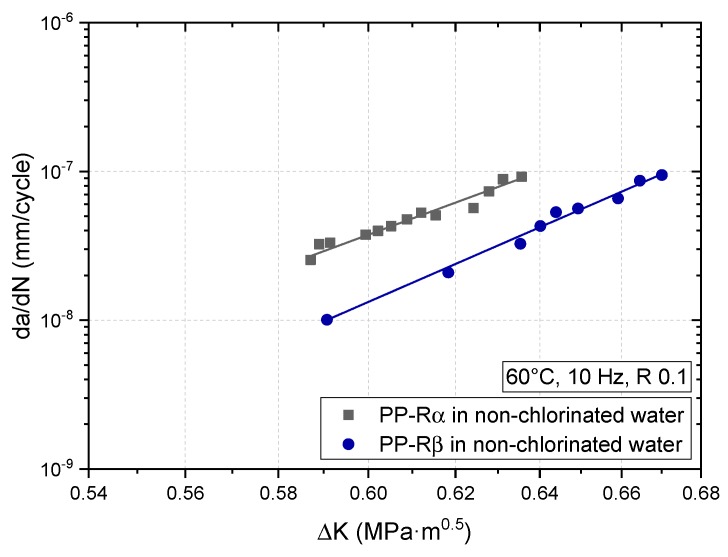
Crack growth curves of monoclinic α crystal type PP-Rα and trigonal β crystal type PP-Rβ tested at 60 °C in non-chlorinated water with 0 mg/L free chlorine.

Fatigue crack growth (FCG) curves of PP-Rα and PP-Rβ for 60 °C in non-chlorinated water (0 mg/L free chlorine) are depicted in Figure 6. PP-Rβ clearly outperforms PP-Rα, which is indicated by much higher crack growth rates over the whole range of ΔK. The smaller spherulite size, the lower packing density, and the favorable arrangement of beta-lamellae inducing a higher degree of mobility of the crystalline and the amorphous phase [1,4] along with a potential phase transformation from β to α in the plastic zone at the crack tip [4], may help to explain the superior FCG resistance of PP-Rβ. While further studies are needed, in our opinion, a crucial factor for the enhanced crack growth resistance of PP-Rβ is certainly attributed to the spherulite structure with its intrinsic higher density of trans-spherulitic tie molecules and inter-spherulitic entanglements.

**Figure 7:**
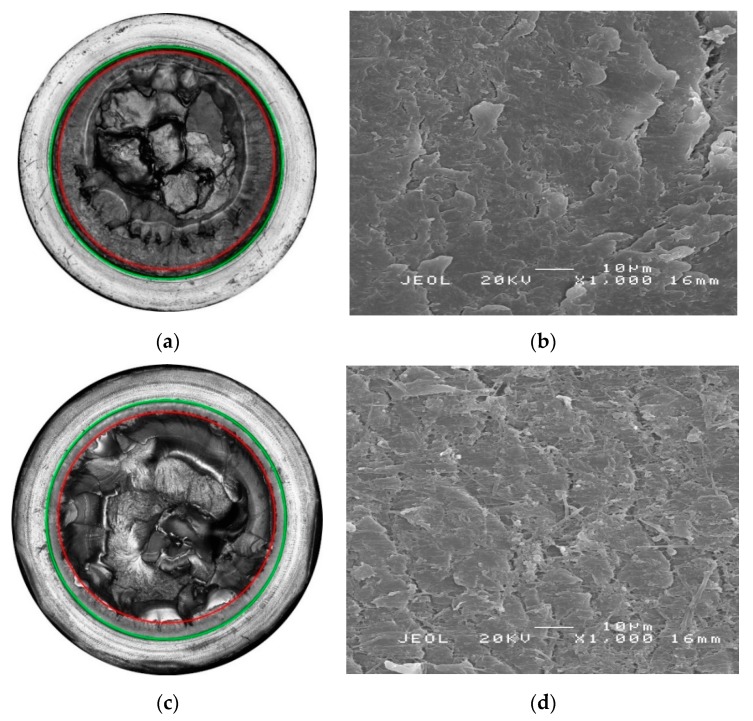
Surface with emphasized start (green circle) and end (red circle) of stable crack growth together with a detail of the stable crack growth region for (**a**,**b**) PP-Rα and (**c**,**d**) PP-Rβ tested at 60 °C in non-chlorinated water with 0 mg/L free chlorine.

Figure 7 illustrates laser scanning confocal microscope (LSCM) images of the fracture surface cross-sections together with scanning electron microscope (SEM) images of fracture surface details in the quasi-brittle crack and stable growth regions for PP-Rα, and PP-Rβ tested at 60 °C in non-chlorinated water (0 mg/L free chlorine). In Figure 7a,c, the beginning and the end of stable crack growth are indicated by green and red circles, respectively. For PP-Rβ longer crack lengths before ultimate failure were obtained. This is shown by the regions between the green and the red circles. It is worth mentioning that both PP-R grades revealed rather short stable crack growth regimes compared to polyethylene pipe grades [46,47]. When considering a polar coordinate system on the LSCM images in Figure 7a,c, the SEM images in Figure 7b,d were taken at 0°. In the latter images, a more finely grained and partially fibrillary structure can be seen for PP-Rβ.

**Figure 8:**
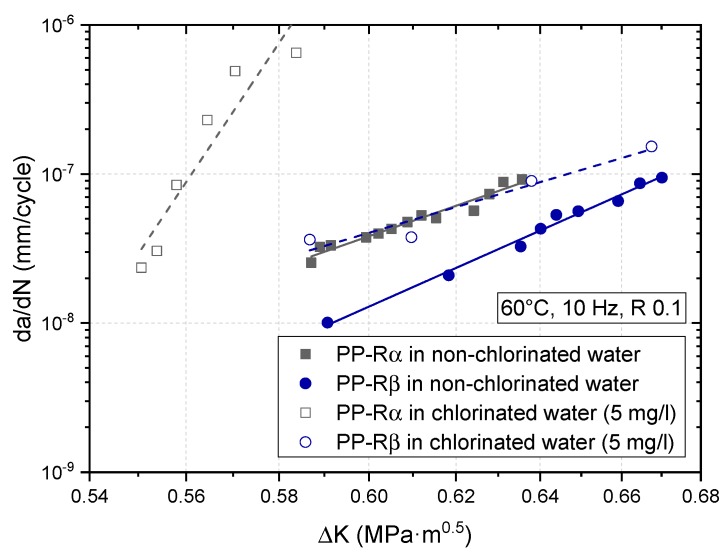
Crack growth curves of PP-Rα and PP-Rβ tested at 60 °C in non-chlorinated water with 0 mg/L free chlorine and in chlorinated water with 5 mg/L free chlorine.

In Figure 8, the FCG curves of PP-Rα and PP-Rβ tested at 60 °C in non-chlorinated water and chlorinated water with 5 mg/L free chlorine are compared. The deteriorating effect of chlorine content on the FCG resistance is quite evident. For PP-Rα, the inferior FCG resistance due to chlorine is shown by significantly higher crack growth rates and lower ΔK values. Conversely, the chlorine-induced decrease in FCG resistance of PP-Rβ leads to an FCG curve, which is comparable to the FCG curve of PP-Rα tested in non-chlorinated water. Both curves, PP-Rα in non-chlorinated water and PP-Rβ in chlorinated water, cover similar ranges of crack growth rate and ΔK. Hence, the crack growth behavior of the beta-nucleated PP-R grade in the more severe environment with 5 mg/L free chlorine is apparently as good as PP-Rα in non-chlorinated water. However, for both material grades, the presence of chlorine in water results in an inferior FCG resistance due to the higher oxidative nature of this medium. This observation is in good agreement with the hypothesis of increased local aging at the crack tip [14,32,33,48], which is probably caused by locally enhanced stabilizer leakage/consumption, and by simultaneous molecular degradation (bond rupture) due to the presence of chlorine.

**Figure 9:**
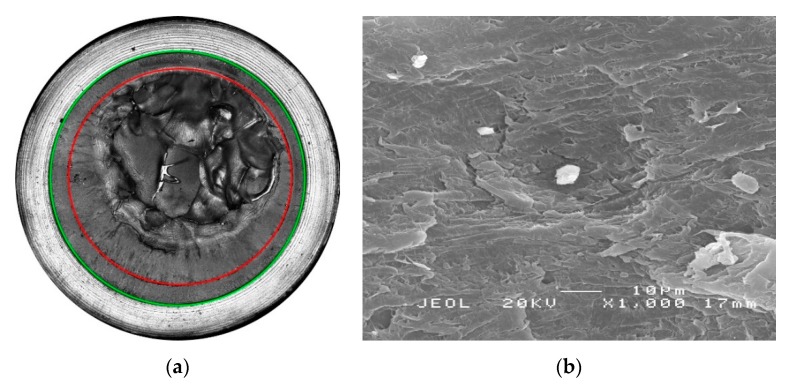

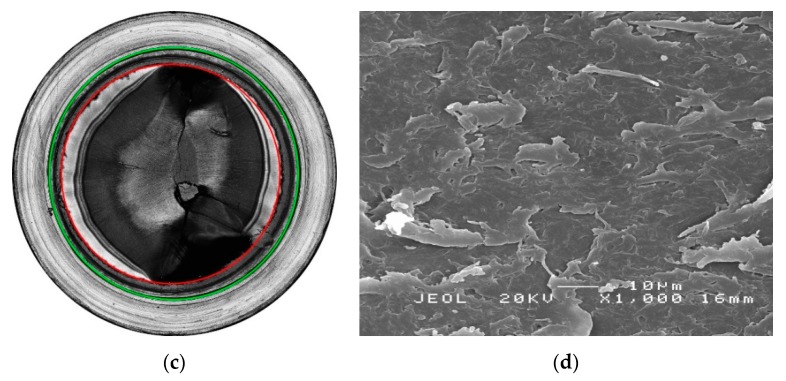
Fracture surface with emphasized end of stable crack growth (red circle) together with a detail of the stable crack growth region for (**a**,**b**) PP-Rα and (**c**,**d**) PP-Rβ tested at 60 °C in chlorinated water with 5 mg/L free chlorine.

Analogous to Figure 7, the fracture surfaces of the CRB specimens of the two PP-R grades tested at 60 °C in chlorinated water (5 mg/L free chlorine) are depicted together with details of the quasi-brittle crack growth region in Figure 9. Again, the stable crack growth is indicated by the area between the outer green circle and the inner red circle (Figure 9a,c). Here too, the SEM images of fracture surface details were taken at 0° when considering a polar coordinate system on the cross-section of the fracture surface (Figure 9b,d). In both SEM images, no fibrillary structures are visible. The lack of fibrils is presumably due to the liquid medium and its highly aggressive nature, which leads to increased aging in the crack tip region and acts to truncate post-yield plastic deformation in the highly deformed crack tip process zone.

## 4. Discussion

In the discussion of the above results, we would like to address two main aspects. First, we will compare and interpret some of our main results with results by others in the literature from a purely phenomenological perspective. Second, we will try to look at our results from a more fundamental perspective in terms of the interdependence of performance properties such as the strain to break and the mean molar mass of our materials, as it is affected by aging exposure.

Grabmann et al. [7] investigated the aging behavior of alpha- and beta-nucleated polypropylene random copolymers in hot air. This environment was chosen based on previous studies by this group, where hot air was shown to be more severe in terms of aging of PP compared to hot non-chlorinated water [49,50,51,52]. In the study, micro-sized specimens of the ethylene co-monomer based PP-R grades were exposed to hot air at five different temperatures ranging from 95 to 135 °C. As expected, the time-to-embrittlement values in hot air were found to strongly depend on the exposure temperature, with time-to-embrittlement values strongly decreasing with increasing temperature. For example, at 95 °C embrittlement times of higher than 15,000 h were determined, while at the highest exposure temperature of 135 °C, total embrittlement was obtained for both grades after about 3300 h. By comparison, the present study revealed time-to-embrittlement values for PP-Rα and PP-Rβ at 60 °C in chlorinated water of 1500 and 2000 h, respectively, both values being significantly below the numbers reported by Grabmann et al. [7] for 95 °C and even 135 °C in air. Clearly, hot chlorinated water is a much more aggressive environment than hot air, with polymer degradation occurring even at low temperatures after shorter exposure times. Simultaneously, Grabmann et al. [7], as well as this study, revealed PP-Rβ as the material with improved aging resistance. The aggressive nature of chlorine in the liquid environment was also corroborated by the mechanical performance investigations, where PP-Rβ was found to outperform PP-Rα both in terms of tensile behavior after environmental exposure and in fatigue crack growth performance under superimposed mechanical-environmental loading. In our opinion, the improved performance of PP-Rβ is not only caused by the higher initial stabilizer content of 0.83 m% compared to 0.63 m% in PP-Rα but primarily by the nucleation-induced different crystalline morphology of PP-Rβ. The latter is characterized by the smaller size of the beta-spherulites [1,4], which for nearly equivalent mean molar masses of unexposed PP-Rα and PP-Rβ implies a higher density of trans-spherulitic tie molecules and inter-spherulitic entanglements.

Turning to the dependence of material performance properties on the molar mass, two basic laws are frequently referred to in polymer science, one being a saturation law [53,54], the other being a power law [54,55]. Transforming the underlying considerations for these laws to the present investigation, which utilized strain at break as the relevant performance property, is complicated by at least three factors, the effect of which on these laws is not yet known. First, it is not quite clear yet, whether strain at break including significant post-yield deformations is to be described better by saturation or a power law. Second, the molar mass changes observed under the present aging conditions of the semi-crystalline PP-R materials investigated are believed to occur primarily in the amorphous and inter-spherulitic regions, whereas the molar mass characterization of the exposed materials provides an integral picture of the entire bulk polymer and thus includes both amorphous and semi-crystalline domains. The latter aspect prohibits any direct correlations on strain at break values on the relevant portion of the molecular structure in the amorphous and inter-spherulitic domains, which are believed to be actually of prime significance in any alterations of strain at break values. Third, the original saturation and power laws for the molar mass dependence of properties were based on polymers with monomodal molar mass distributions. In contrast, the two-phase morphology model of semi-crystalline polymers with molecular aging taking place primarily in the amorphous and inter-spherulitic domains may transform the original monomodal molar mass distribution into a bimodal or multimodal molar mass distribution as advanced aging takes place, for which evidence on which polymer physical law to use is still lacking.

**Figure 10:**
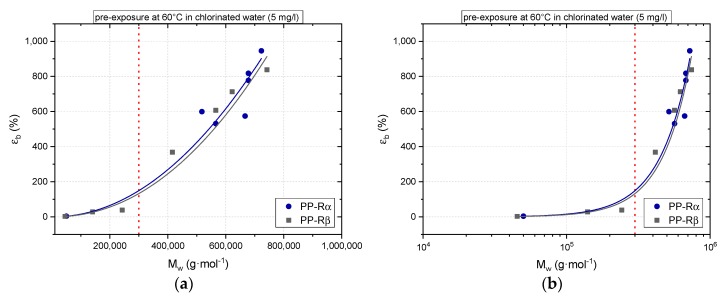

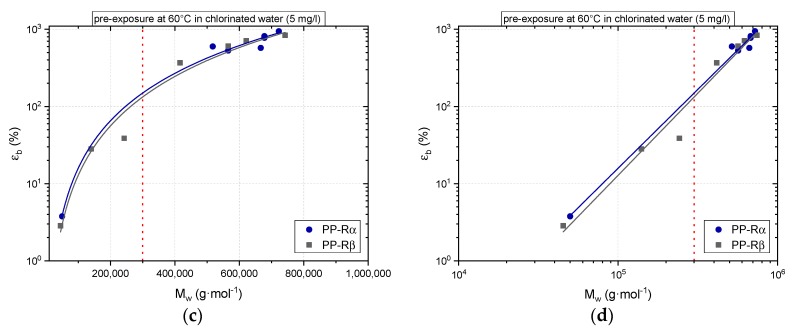
Dependence of strain at break values (ε_b_) of aged PP-Rα and PP-Rβ on the mean molar mass (*M*_w_) of the materials in the aged state comparing diagrams of various scales; (**a**) linear ε_b_ vs. linear *M*_w_, (**b**) linear ε_b_ vs. log *M*_w_, (**c**) log ε_b_ vs. linear *M*_w_, and (**d**) log ε_b_ vs. log *M*_w_.

Considering these fundamental uncertainties, we have chosen in Figure 10 to depict our strain at break values as a function of mean molar mass in four different diagrams interrelating the two quantities in different combinations of linear and log scales. Interestingly, and independent of the scales used, both materials seem to exhibit rather similar behavior when using a power law fit function for the individual data sets. Moreover, there seems to be a characteristic value for the mean molar mass of about 300 kg/mol (see red dotted line in Figure 10) below which quasi-brittle fracture with strain values at around the yield strain value of virgin materials is rapidly approached. This is more readily apparent in Figure 10a–c but is truncated in the log-log diagram in Figure 10d. The mean molar mass value compares quite well with studies by Wallner et al. [56], who investigated the aging behavior of PP-Rβ in hot air at 135 °C and reported a critical mean molar mass value of 330 kg/mol for material embrittlement. In another study by Fayolle et al. [57] of unstabilized PP aged again in air in the temperature range from 70 to 130 °C, characteristic (“critical”) molar masses between 150 and 230 kg/mol were deduced. While these comparisons seem to indicate a critical mean molar mass threshold for PP, which is independent of aging conditions, below which quasi-brittle failure occurs, further work is needed to substantiate this finding and to elucidate the molecular mechanistic model to explain the relationship between strain at break and mean molar mass for aged polypropylene.

## 5. Summary and Conclusions

The influence of hot chlorinated water on material aging, mechanical tensile performance and fatigue crack growth (FCG) resistance was investigated for two different polypropylene random copolymer (PP-R) grades. One PP-R exhibited a monoclinic crystal form (PP-Rα), the other was beta-nucleated with a trigonal crystal form (PP-Rβ). Exposure of micro-sized specimens to chlorinated water with 5 mg/L free chlorine at 60 °C resulted in continuous aging and material degradation indicated by a decrease in stabilizer content, a decrease in onset oxidation temperature, a decrease in mean molar mass, an increase in carbonyl index, and a decrease in mechanical strain at break.

When comparing PP-Rα and PP-Rβ, our experimental results clearly indicate that the latter material grade in its structural performance under chlorinated water exposure is superior and hence to be preferred. For example, for PP-Rα total embrittlement was obtained after 1500 h, whereas PP-Rβ exhibited a time to embrittlement of 2000 h. The higher stability of PP-Rβ is probably due to the slower antioxidant consumption caused by the fine spherulite structure in PP-Rβ. Moreover, in non-chlorinated water at 60 °C, for PP-Rα a significantly reduced FCG resistance was obtained compared to results for PP-Rβ. For both PP-R grades, an increase in chlorine content from 0 to 5 mg/L free chlorine resulted in a decrease in FCG resistance indicated by higher crack growth rates for a given stress intensity factor range. This behavior is most likely caused by the enhanced oxidative nature of chlorinated water, which also acts in terms of local aging at the crack tip. Nevertheless, the FCG resistance for PP-Rβ in chlorinated water essentially turned out to be equivalent to the FCG resistance of PP-Rα in non-chlorinated water, at least over the crack growth rate range investigated.

Finally, when analyzing and interpreting the effects of aging exposure on alterations in the mean molar mass and then further on the strain at break values, a characteristic value for the mean molar mass of about 300 kg/mol was determined below which quasi-brittle fracture occurs. For both materials investigated, this characteristic value of mean molar mass indicating significant material degradation was more rapidly reached in the chlorinated water environment, while in the non-chlorinated water environment no such degradation could be observed in a comparable test time. While the characteristic mean molar mass value for the onset of quasi-brittle failure is in good agreement with other investigations in the literature, on a finer scale numerous questions remain as to the influence of morphological effects (i.e., degree of crystallinity, spherulite size distribution, etc.) on the resulting multimodal molar mass distribution and the kinetics of mean molar mass degradation. Assuming that molecular degradation under aging exposure conditions primarily takes place in the amorphous inter-lamellar and inter-spherulitic regions, it is not a priori clear whether the dependence of the strain at break on the mean molar mass for newly synthesized polymers and aging-degraded polymers are in fact equivalent. In other words, a different characteristic mean molar mass value for quasi-brittle failure may perhaps exist depending on the modality of the molar mass distribution as well as on whether the same molecular mass distribution is achieved by a newly synthesized polymer, which is subsequently processed and investigated, or a polymer for which this equivalent mass distribution is achieved from a processed and exposed polymer by aging.

## Figures and Tables

**Table 1:** Material designation, crystal phase, and pipe material classification.

Material Designation	Crystal Phase	Pipe Material Classification
PP-Rα	Monoclinic (α)	PP-R: PP random copolymer
PP-Rβ	Trigonal (β)	PP-RCT: PP random copolymer with a special crystalline morphology and improved pressure and temperature resistance
